# Evolution of Open Magnetic Flux and Heliospheric Current Sheet-Associated Streamer Wind Across Multiple Solar Cycles

**DOI:** 10.1007/s11207-026-02664-8

**Published:** 2026-05-15

**Authors:** Jackson R. MacTaggart, Liang Zhao, Susan T. Lepri, Lennard A. Fisk, Aidan J. Nakhleh

**Affiliations:** Department of Climate and Space Sciences and Engineering, University of Michigan, Ann Arbor, MI USA

**Keywords:** Solar cycle, Solar wind, In-situ measurements

## Abstract

With the advent of Ulysses measurements during the solar minima of Solar Cycles 23 and 24, the heliospheric magnetic field and the solar wind were observed to behave much differently than expected. In particular, previous studies showed that the magnetic open flux of the Sun, calculated as the product of the radial component of the Sun’s magnetic field and the squared radial distance from the Sun, was observed to have decreased along with observed changes in solar-wind streams, such as solar wind proton density. This was in contrast to the standing theory, prior to the measurements made by Ulysses, that the “baseline” of the magnetic open flux should remain constant across the minima. Studies conducted after these measurements accounted for the discrepancies by showing that the Total Open Magnetic Flux (TOMF), the total amount of open flux outside the streamer belt, is a conserved “baseline” quantity from solar minimum to minimum. In this work, we examine a range of solar-wind parameters across five solar minima in Cycles 21, 22, 23, 24, and 25. Using measurements of the heliospheric magnetic field, dynamic solar wind, and heavy ion composition from multiple spacecraft, we investigate the width of the Heliospheric Current Sheet (HCS) streamer belt in the recent Solar Cycle 25 solar minimum using Advanced Composition Explorer (ACE) Solar Wind Ion Composition Spectrometer (SWICS) (1.1 and 2.0) and Solar Wind Electron, Proton and Alpha Monitor (SWEPAM) data. We also investigate the width of the HCS-streamer belt for the Solar Cycle 22 minimum using a newly proposed entropy methodology. With this analysis, we show continued validation of the conservation theory for the TOMF at solar minimum for Solar Cycles 22 and 25.

## Introduction

### The Open Magnetic Flux

Under the Parker model of the solar wind, the open magnetic field of the Sun extends radially away from the Sun due to it being dragged outwards by the supersonic solar wind, in addition to Gauss’s law for magnetism ($\nabla \cdot \boldsymbol{B} = 0$) requiring open field lines to continuously extend outward and loop back to regions of opposite polarity out into the solar system and beyond (Parker 1958). We define the “closed” magnetic flux as those magnetic-field lines that stay attached to the Sun at both ends. Closed field lines are taken to be below the 2.5 solar radii surface defined by potential field source surface models (Wang and Sheeley 1992). In turn, we define the “open” magnetic flux as those magnetic-field lines with one end attached to the Sun and the other end that extend outwards for some vast distance past the 2.5 solar radii boundary. Open solar magnetic-field lines are carried outward by the solar wind to form the heliospheric magnetic field, which extends throughout the heliosphere to the heliopause (Owens and Forsyth 2013). Observations from spacecraft such as Ulysses show that throughout the solar cycle open magnetic flux is separated by the Heliospheric Current Sheet (HCS) into two hemispheres of opposing polarity (Balogh and Smith 2001).

At solar minimum, the HCS is observed to be located near the solar equator with relative smoothness (*i.e.*, relatively confined to the ecliptic with minimal warping). Open magnetic flux is generally distributed evenly across the solar corona outside of the HCS. During solar minimum, however, the open magnetic flux at the Sun is observed to be clustered at the poles, which gives rise to the polar coronal holes observed at solar minimum (the open magnetic flux still becomes distributed throughout the heliosphere as it extends out from the Sun). As the solar cycle transitions away from the minimum, the HCS becomes more and more inclined and warped until it returns relatively even at the equator at the next solar minimum. At this point, the polarized hemispheres have been reversed (Hoeksema, Wilcox, and Scherrer 1983; Smith 2001).

A basic property of the open magnetic flux at solar minimum is that it can only be reduced or removed by reconnecting with open magnetic flux of opposite polarity. It cannot be destroyed near the Sun by interacting with the closed loops that define the closed magnetic flux—only displaced and moved in a diffuse manner around the Sun (Fisk 2005). However, this removal by reconnection is unlikely due to the well-defined HCS that separates the two hemispheres of open magnetic flux of opposing polarity. Additionally, there have been no clear observations of this phenomenon occurring. If removal of open flux by reconnection were to happen, we would see electron heat flux dropout near the Sun like we can near Earth, but this has not been observed (Owens, Crooker, and Lockwood 2013). This tells us that the scenario for open magnetic flux being eliminated at solar minimum is unlikely to actually occur (Owens and Forsyth 2013).

Therefore, polarity reversal of the open magnetic flux that occurs from solar minimum to solar minimum means that the open magnetic flux must be transported alongside the warping HCS (Fisk and Schwadron 2001).

Fisk (2005) describes the method of transport as a process where the open magnetic flux interacts with coronal loops, especially small loops (*i.e.*, loops with lengths relatively smaller than supergranules). Reconnection can occur between the open flux and the base of the loop without destroying or creating more open flux. As a result of the reconnection, the open flux line is displaced by the footpoint separation of the loop. This creates a diffusive process by which the open flux moves, in addition to a diffusive process resulting from random convective motions in network lanes. Fisk (2005) derives the footpoint separation distance as $h$ and the mean squared separation of loops as

1 $$ \overline{h}^{2} = \frac{8}{3N_{o}} + \frac{4}{3} \frac{h_{e}^{2}N_{e}\delta t}{N_{l}N_{o}\delta h^{2}}, $$

where $h_{e}$ is the footpoint separation of small loops, $N_{e}$ is the rate of emergence of new magnetic loops on the Sun per unit area, $t$ is time, $h$ is the separation of footpoints, $N_{l}$ is the surface density of loops, and $N_{o}$ is the surface number density of open field lines. Fisk (2005) then derives a diffusion coefficient due to diffusion from convective motion

2 $$ \kappa _{1} = \frac{\delta h^{2}}{2\delta t} $$

and a diffusion coefficient due to reconnection with small loops

3 $$ \kappa _{2} = \frac{\overline{h}^{2}}{4\tau _{c}}, $$

where $1/\tau _{c}$ is the collision frequency of open field lines with loops. Putting everything together, Fisk and Schwadron (2001) derive the motion of open magnetic flux, $B_{\mathrm{o}}$, as

4 $$ \frac{\partial B_{\mathrm{o}}}{\partial t} = \nabla ^{2}(\kappa B_{\mathrm{o}}) - \nabla \cdot (\textbf{u}B_{\mathrm{o}}), $$

where $\textbf{u}$ is the convection velocity at the solar surface from differential rotation and meridional flow and $\kappa $ is a diffusion constant. Fisk (2005) defines $\kappa = \kappa _{1} + \kappa _{2}$.

**Figure 1:**
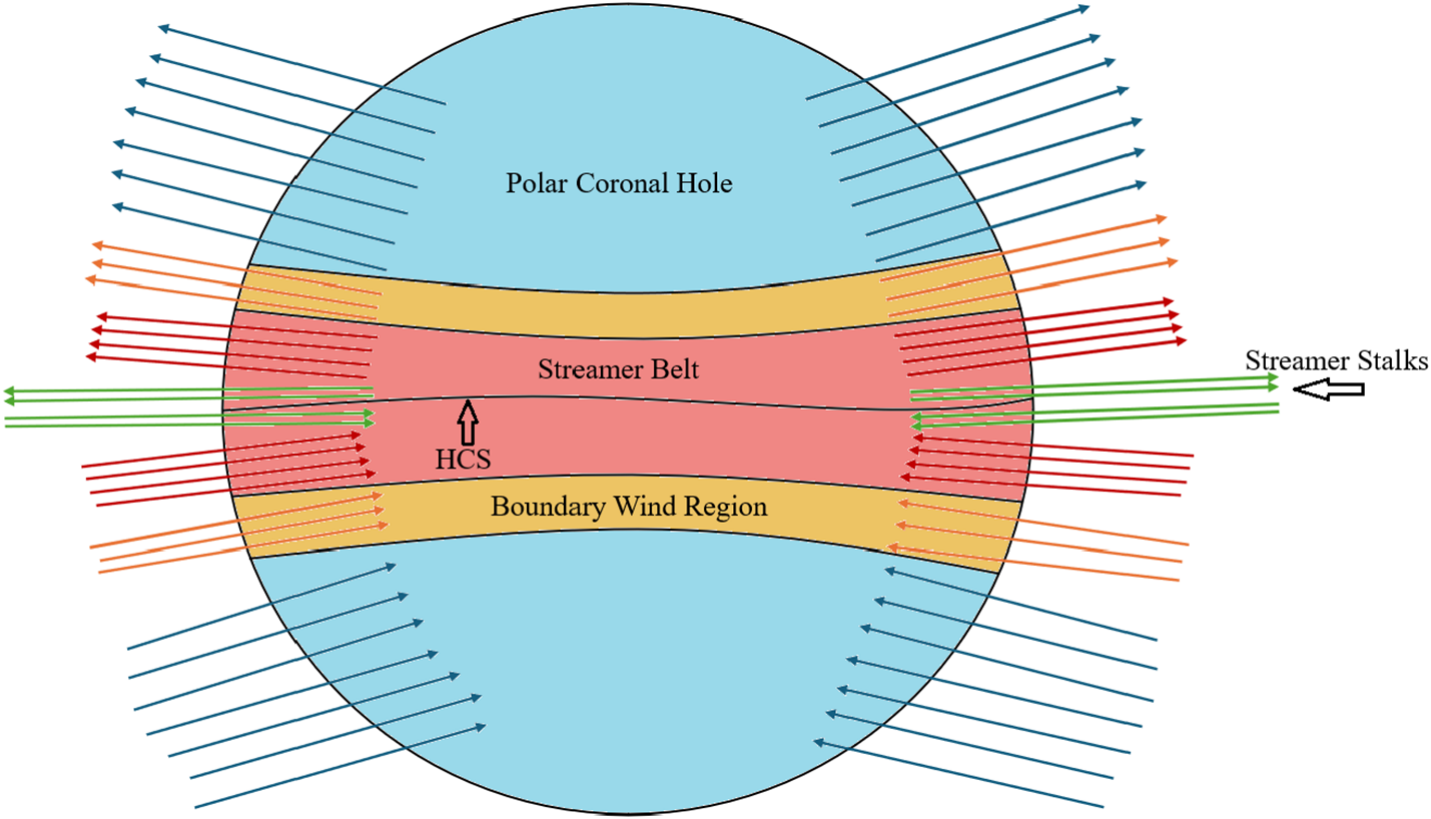
Schematic representation of the global solar corona during solar minimum mapped out to around 2.5 solar radii, illustrating the large-scale magnetic field configuration. At this distance away from the Sun, the magnetic-field lines, represented here by the different colored arrows (with direction indicating outward or inward polarity), are generally considered to be radial. The equatorial region is dominated by the solar streamer belt (red), characterized by open field lines carrying the slow solar wind further out into the heliosphere. Close to the HCS that splits the streamer belt, the open magnetic-field lines taper into the narrow radial extensions of about 1 – 2^∘^ in angular width known as streamer stalks (green). Conversely, the higher-latitude regions are characterized by fast wind from polar coronal holes (blue), which source the open magnetic-field lines at minimum. While closer to the Sun the polar coronal holes are restricted to high latitudes above $\pm 60^{\circ}$, the fast wind extends to lower latitudes farther out into the heliosphere (Harvey and Recely 2002; McComas et al. 2008). The transition region (orange) between the streamer belt region and the polar coronal holes is identified as the boundary region between the fast and slow wind (Ko, Roberts, and Lepri 2018). Features not to scale.

At solar minimum, high-speed streams of solar wind carry the solar magnetic field into the heliosphere, filling it with two regions of opposite polarity separated by the HCS. In between these high-speed streams, dense and slower moving solar wind is present. This smaller region is known as the “streamer belt” (Roberts, Keiter, and Goldstein 2005). At the boundary of this region, the streamers taper into narrow radial extensions about 1 – 2^∘^ in angular width, known as streamer stalks (distinct from the broader 20 – 40^∘^ streamer belt that encompasses them). Because these stalks delineate the HCS, the streamer-belt region is also referred to as the streamer-stalk region (Woo and Martin 1997). Figure 1 shows a schematic detailing these features.

The general consensus in the heliophysics community is that the slow solar wind is mostly related to the streamer belt, whereas the fast solar wind generally originates from coronal holes (Zirker 1977; Schwenn 2006; Zhao, Zurbuchen, and Fisk 2009). Ulysses observations have shown that at solar minimum, the slow solar wind can be widely restricted to low-latitudes near the solar equator around the streamer belt, usually within $\pm 15^{\circ}$ of latitude (Wenzel et al. 1992; Antonucci, Dodero, and Giordano 2000). At solar minimum, the streamer belt is in such a configuration that allows it to be inclined at low latitudes and flattened (*i.e.*, without the warping that can be observed at other points in the solar cycle, especially at solar maximum). Furthermore, despite the fast solar wind being able to approach latitudes of $\pm 30^{\circ}$ out in the heliosphere, polar coronal holes, from which the fast solar wind originates, during solar minimum are largely restricted to high latitudes (above $\pm 60^{\circ}$), allowing us to define the streamer belt further (Harvey and Recely 2002; McComas et al. 2008; Antiochos et al. 2011). Fast and slow solar wind can also be further distinguished by their densities at solar minimum—the slow solar wind having high density similar to the streamer belt and the fast wind and polar coronal holes being of lower density —and composition (McComas et al. 2003). The elemental composition of the solar wind is helpful for identifying the regions from which different solar wind streams originate (Ogilvie and Coplan 1995).

Zhao and Fisk (2011) define the streamer belt by dividing the solar wind into different types. They categorize the solar wind in three ways: non-transient wind originating from inside or outside coronal holes and solar wind associated with transient Interplanetary Coronal Mass Ejections (ICMEs). Here, we define wind outside coronal holes during solar minimum as being solar wind originating from high coronal electron temperature regions in the low to mid latitudes and not in the high latitude, low temperature polar coronal holes (Tokumaru et al. 2009).

Focusing on the non-transient definition for our purposes, Zhao, Zurbuchen, and Fisk (2009) found that there is a distinct threshold between coronal hole and non-coronal hole, or streamer, solar wind, marked by a sharp transition when the ratio of O^7+^/O^6+^ crosses the value of 0.145. The type of solar wind observed is strongly dependent on the composition of its origins in the corona. This is because the charged particles that make up the solar wind have different freeze-in points, so ionic charge composition in turn becomes a direct indication of the coronal temperature from which it originated (Hundhausen, Gilbert, and Bame 1968; Zhao, Zurbuchen, and Fisk 2009). Close to the Sun, the solar wind is hot and dense, which allows for ions to have high rates of ionization and recombination with electrons. This means that close to the Sun, the charge state composition of ions is an evolving parameter. As the solar wind accelerates away from the Sun, its density decreases, reducing ionization and recombination events. In turn, temperature becomes less effective at changing charge state composition. Eventually, the solar wind “freezes in” and causes the charge state to become locked. Different locations and phenomena on the Sun that act as origins for the solar wind “give” it different temperatures and densities (and, therefore, freeze-in points within a few solar radii from the solar photosphere), so composition is a critical parameter for identifying the type of solar wind being observed (Landi et al. 2012).

At solar minimum, composition measurements have consistently shown that solar wind origins are distinguished well by the ratio of O^7+^/O^6+^ because of said ratio’s fast freeze-in process in the low corona (Zurbuchen et al. 2002). The delineation between fast and slow wind when the ratio of O^7+^/O^6+^ is 0.145 has been verified through a few different methods, as observed in Figure 3.

**Figure 2:**
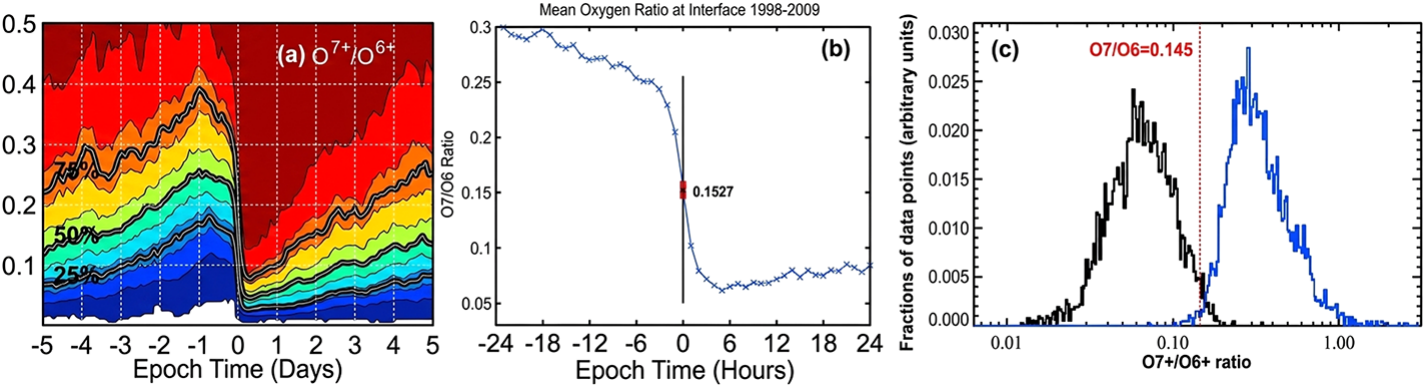
(a) Cumulative distribution function for 258 stream interfaces showing a distinct drop in the ratio of O^7+^/O^6+^ at each interface (used with permission from Crooker and McPherron 2012). (b) Epoch analysis showing that the interface criterion for separating fast and slow wind is essentially that of the 0.145 value shown in Zhao, Zurbuchen, and Fisk (2009) (used with permission from Crooker and McPherron 2012). (c) Well-separated fast and slow solar wind around the ratio of O^7+^/O^6+^ being 0.145, as found through the application of machine learning (used with permission from Zhao et al. 2024).

In Figure 2(a), Crooker and McPherron (2012) measured 258 solar wind stream interfaces (the boundaries between fast and slow wind) as measured by the Advanced Composition Explorer (ACE) using cumulative distribution functions (Gloeckler et al. 1998). When the interface is reached at the point marked 0 in Epoch Time, there is a sharp drop in the ratio of O^7+^/O^6+^, which corresponds to a sharp increase in solar wind speed (see Figure 2(a) and Crooker and McPherron (2012)). Similarly, in Figure 2(b) here, and from the same work, averaging the value at which the solar wind switches from fast wind to slow wind at the interface from Figure 2(a) produces a O^7+^/O^6+^ of 0.15, which is almost the same as, and ostensibly is, the 0.145 value reported by Zhao, Zurbuchen, and Fisk (2009). Finally, we see in Figure 2(c) that when Solar Orbiter solar wind and charge state composition data is fed into machine learning models, the models show that different types of solar wind are well-separated around the same value of 0.145 in the O^7+^/O^6+^ ratio (Zhao et al. 2024).

In this manner, slow solar wind being defined by the ratio of O^7+^/O^6+^ being greater than 0.145 can also define the streamer wind at solar minimum, if both the slow wind and streamer belt are observed at such low latitudes. Zhao and Fisk (2011) later verified this relationship to be true with observations.

A longstanding theory in solar physics concerning the open magnetic flux ($|B_{r}|r^{2}$) at solar minimum prior to the Ulysses measurements discussed in Smith and Balogh (2008) was that the solar minimum should represent the “baseline” level of activity for the Sun and the open magnetic flux should remain constant across the minima. However, with the advent of non-ecliptic measurements from Ulysses, the value of open magnetic flux at solar minimum was instead found to vary. In fact, from the Cycle 23 minimum to the Cycle 24 minimum, the open magnetic flux was found to drop by about 20% (as we calculate and show later in Table 1). For a quantity that is supposed to remain constant (or relatively so) from solar minimum to solar minimum, this is a large change and a contradiction to the aforementioned standing theory.

**Table 1:** Length of solar minimum and average $\langle |B_{r}| r^{2}\rangle $ at solar minimum for Cycles 21 – 25. From solar cycle to solar cycle, the value of open magnetic flux at solar minimum is never constant - rather it changes and sometimes by drastic amounts, as was found in Smith and Balogh (2008).

Cycle	Length of Solar Minimum (years)	$\langle |B_{r}| r^{2}\rangle $ (nT AU^2^)	Percent Change
21	4.0	3.54	—
22	5.0	4.01	13.13
23	4.5	3.21	−19.87
24	5.5	2.54	−20.64
25	5.0	2.81	10.32

Because of this deviation from expectation, a better way to understand the reduction in observed open magnetic flux comes from the work of Zhao and Fisk (2011). This work proposed and validated (for Solar Cycles 23 and 24) a new theory to describe how open magnetic flux is indeed conserved. It also proposed a new quantity called total open magnetic flux (TOMF) that is conserved from solar minimum to solar minimum:

5 $$ \mathrm{TOMF} = \Omega _{\mathrm{OSB},1}|B_{r}|_{1}r_{1}^{2} = \Omega _{\mathrm{OSB},2}|B_{r}|_{2}r_{2}^{2} $$

Here, $\Omega _{\mathrm{OSB}}$ is defined to be the solid angle of the region outside of the streamer belt (OSB) where open flux is observed and is measured at 2.5 solar radii. $|B_{r}|r^{2}$ is the observed open magnetic flux. Simply put, this equation tells us that the product of these two parameters at one solar minimum will be the same at any other solar minimum because TOMF must still be conserved.

Reconciling the conservation of TOMF and open magnetic flux (*i.e.*, reasoning why we need to include the extra parameter of $\Omega _{\mathrm{OSB}}$) requires only minor modifications to the previous understanding of open magnetic flux. Previous explanations of the conservation of open magnetic flux like Svalgaard and Cliver (2007), later modified and expanded on in 2011, 2024 and 2025, maintained the idea of a floor of open magnetic flux from minimum to minimum carried outwards by the slow solar wind (Cliver and Ling 2011; Cliver, White, and Richardson 2024; Cliver, Richardson, and Martin 2025). As a result of the floor appearing to be breached during the 2008 – 2009 solar minimum due to the observed drop in open magnetic flux, Zhao and Fisk (2011) built on the previous theory and introduced the $\Omega _{\mathrm{OSB}}$ parameter to suggest that the floor wasn’t breached in terms of total flux, just redistributed geometrically. This additional line of thinking stays true to the underlying understanding of why TOMF must be conserved (*i.e.*, open flux cannot be destroyed through reconnection with closed magnetic flux, only by reconnection with open flux of opposing polarity (Fisk and Schwadron 2001)). Observational evidence for the solar minima of Cycles 23 and 24 that included $\Omega _{\mathrm{OSB}}$ confirmed the idea of conservation of TOMF (Zhao and Fisk 2011).

In this article, we continue to validate this theory for the newest solar minimum in Solar Cycle 25, as well as the past solar minimum in Solar Cycle 22. Section 2 lays out the observational methodology we employed, including the use of in-situ magnetic field data from Ulysses, compositional data from the ACE Solar Wind Ion Composition Spectrometer (SWICS), etc., essential for achieving our research goals (Gloeckler et al. 1998). Section 3 presents the primary outcomes of our analysis, highlighting our validation efforts and new research implications that have arisen from them. Section 4 summarizes our methodology and results while also considering additional research that still needs to be pursued.

## Observations

### Measuring the Sun’s Open Magnetic Flux Across Solar Cycles

We examined the open magnetic flux measurements, defined as the product of the absolute value of the in-situ measurements of the radial component of the Sun’s magnetic field and the squared radial distance from the Sun ($|B_{r}|r^{2}$), from six different spacecraft (ACE, Solar Orbiter, Geotail, International Sun-Earth Explorer-3 [ISEE-3], Interplanetary Monitoring Platform-8 [IMP-8], and Wind) at L1 or closer across five solar minima in Cycles 21, 22, 23, 24, and 25, with special focus on the latter three minima (Scearce et al. 1976; Durney and Ogilvie 1978; Nishida et al. 1992; Lepping et al. 1995; Mueller et al. 2013). As discussed in Section 1, Ulysses measurements confirmed that from the Cycle 23 solar minimum to the Cycle 24 solar minimum, the open magnetic flux dropped (Smith and Balogh 2008). We extended our initial analysis of open magnetic flux to Cycles 21, 22, and 25 to confirm that this was not a fluke measurement. Measuring open magnetic flux from only one solar cycle to another makes for a poor statistical sample, so extending our analysis to these additional solar cycles gives additional observational evidence while simultaneously improving our understanding of what is occurring. This extension builds on our goal of validating the Zhao and Fisk (2011) theory for the most recent solar cycle minimum.

To account for the different polarities of $B_{r}$ measured at any given time, we followed the process of Smith (2011) and measured $|B_{r}|r^{2}$ instead of just $B_{r}r^{2}$. Using the modulus allows us to better cope with the magnetic sector structure of the Sun, especially when averaging. In taking a raw average, we could sum and average positive and negative values of $B_{r}r^{2}$ of the same magnitude, which would just cancel each other out and skew our results. Thus, taking the absolute value of the open magnetic flux first is preferred and results in a more accurate interpretation of what is being observed.

Because we are interested in examining the TOMF at solar minimum, we need to define when solar minimum is - or what time period it covers. We define absolute solar minimum to be at the absolute minimum of sunspot number in a given solar cycle. This is a hallmark of the solar/sunspot cycle and the correlation between the two that uses the sunspot number to tell which phase of the solar cycle is happening at a given time.

Unlike the sunspot number, the strength of the open magnetic flux is not so strictly bound to the solar cycle. It follows the same sinusoidal pattern of the solar cycle (growing in strength near solar maximum and decreasing in strength at solar minimum), but the chaotic nature of the Sun means that even at the “baseline” activity level of solar minimum, the weakest measurement of open magnetic flux might come at a time other than the absolute solar minimum as we have defined it here. Instead, we measured the average open magnetic flux across a defined solar minimum interval, chosen based on sharp rates of change in sunspot number (indicating, in turn, a sharp rate of change in the strength of the solar cycle). Given that the sunspot number is a direct measurement for how strong or weak the solar cycle is at a given moment, the absolute minimum of the sunspot number in a given cycle tells us, in turn, the moment of the absolute solar minimum of the solar cycle. In Table 1, we find that the unique time periods for each solar cycle around this point are both short enough not to broach the down- or up-cycle periods and long enough to cover the whole minimum. We find divergences in open magnetic flux values when we go outside or inside these time periods.

**Figure 3:**
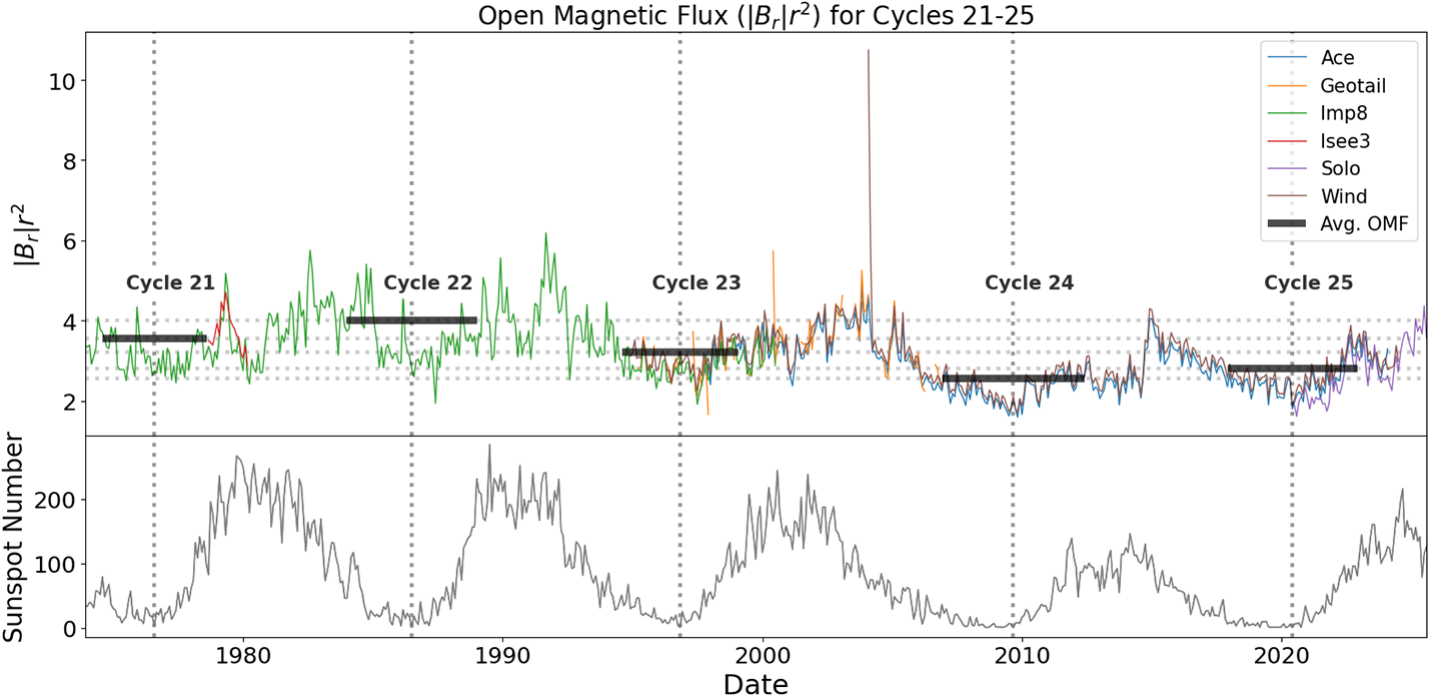
The top panel of Figure 3 shows monthly-averaged $|B_{r}|r^{2}$ for IMP-8, ISEE-3, Wind, Geotail, ACE, and Solar Orbiter. from 1973 to present day. The bottom panel shows monthly averaged sunspot number data for the same time period. The vertical dotted line shows what we define as the absolute solar minimum according to what the absolute sunspot number minimum value is for a given labeled solar cycle. The horizontal gray lines show which open magnetic flux data was considered for each solar minimum time period. Finally, the horizontal gray lines show that the average open magnetic flux at solar minimum changed from solar minimum to solar minimum and help to show by how much the open magnetic flux is different at one minimum versus another.

Our open magnetic flux measurements can be seen in Figure 3. We can clearly see that the open magnetic flux decreases from Cycle 23 to 24 and then slightly rises from Cycle 24 to 25. The actual values of the average open magnetic flux can be seen in Table 1.

Table 1 shows the average open magnetic flux value at each solar minimum from Cycles 21 – 25. The values in the second column are the exact values corresponding to their respective black bars in Figure 3. Not only are these the values used in calculating the TOMF for a given solar minimum, but they serve a second purpose here in showing the exact percent change by which open magnetic flux has changed from solar minimum to solar minimum. This confirms that the original Ulysses measurements of decreasing open magnetic flux from the Cycle 23 minimum to the Cycle 24 minimum were not fluke measurements.

For the purpose of measuring *total* open magnetic flux, we want to make sure that we are measuring *open* magnetic in the non-streamer region only. However, for measurements taken at Earth, instruments cannot solely measure open magnetic flux. As shown in Crooker et al. (2004), in-situ measurements away from the Sun, closer to Earth, and within the streamer belt surrounding the HCS can have, through interchange reconnection, local magnetic field reversals (now called magnetic switchbacks) - regions of opposing polarity from the same field lines embedded within the interplanetary magnetic field. When we measure $|B_{r}|r^{2}$, we average across time intervals of length dependent on the activity of the given cycle (*i.e.*, when activity cycles begin and end). This all means that equatorial spacecraft measure the radial magnetic field of both open flux and loops. Due to the curvature of magnetic loops close to the Sun, we expect the radial magnetic field to decrease from its value at being fully radial within loops simply due to the curvature of loops. In other words, the radial magnetic field we measure in the loops is inherently going to be smaller than that of purely open flux and needs to be accounted for. Fortunately, we do not have to worry about this for out-of-ecliptic measurements from spacecraft like Ulysses, as such measurements are very clear when we are inside or outside of the streamer belt. In turn, we can use this fact to correct measurements at Earth using Ulysses measurements at the same time periods to correct for these local field reversals. We find that this correction factor is approximately 1.14, following the process of Fisk et al. (2026).

### Investigating the Width of the HCS-Streamer Belt

To determine the total solid angle of the coronal hole, one could naively subtract the HCS streamer belt angular extent from $4\pi $ steradians. However, for a more robust estimate we leverage in-situ measurements of solar wind composition to quantify the region in the heliosphere occupied by the streamer belt wind/slow wind. However, in practice this is nontrivial. To measure the HCS-streamer belt width, we first need to identify streamer wind. We do this by using data from ACE. In particular, we want data taken by SWICS. Because we want to identify the streamer wind, we follow the work of Zhao and Fisk (2011) discussed in Section 1 and utilize the O^7+^/O^6+^ solar wind ratio data.

Before we proceed with discussing our method of using composition to identify the slow wind, we must discuss a change in the operational state of SWICS that affects collected data. On 23 August 2011, a radiation induced anomaly in the hardware cause a degradation in the SWICS instrument, changing how it took data. Under certain charge state composition ranges, the SWICS data has become saturated to the point it could no longer be used. Because of this, SWICS data is separated into pre- and post-degradation datasets, SWICS 1.1 and SWICS 2.0, respectively. Generally, because of the degradation issue, one must take care in interpreting the SWICS 2.0 data by applying a filter to take out the saturated data before using it.

**Figure 4:**
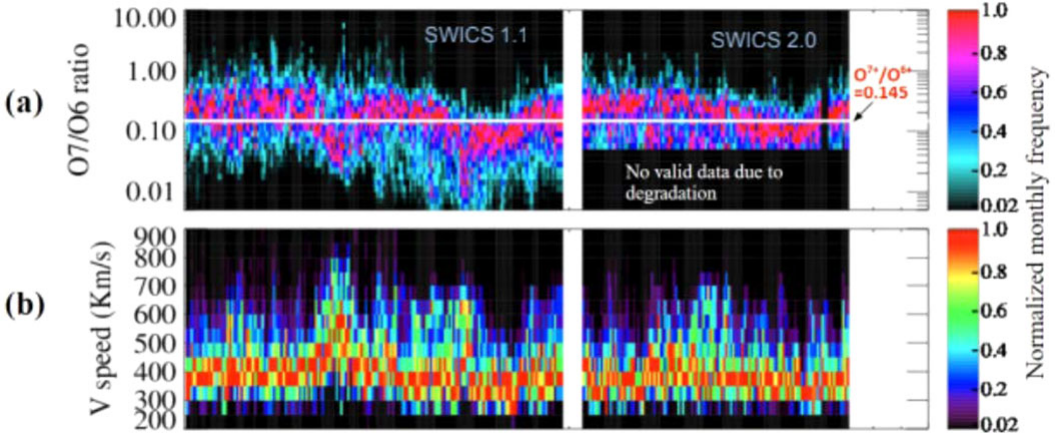
(a) Frequency of O^7+^/O^6+^ normalized by month as measured by ACE/SWICS. (b) Proton speed as measured by ACE/SWEPAM.

However, this is not the case with the O^7+^/O^6+^ data we examine, because, fortunately, this ratio at and above 0.145 was largely unaffected by the degradation. Therefore, we can use it with a high level of trust to find the streamer belt. In particular, we look for when the O^7+^/O^6+^ ratio is greater than 0.145, as this ratio range is indicative of streamer wind. This is shown in Figure 4. Figure 4(a) shows the frequency of O^7+^/O^6+^ measurements normalized by month as measured by ACE/SWICS. We utilize measurements above the white horizontal line, which marks where O^7+^/O^6+^ is above 0.145. Figure 4(b) shows the proton speed normalized by month as measured by the ACE Solar Wind Electron, Proton and Alpha Monitor (SWEPAM) (McComas et al. 1998). The vertical white bars in (a) and (b) mark the separating event between the SWICS 1.1 and SWICS 2.0 datasets, which we discuss next. We will show next that for all of the SWICS data we are interested in for a given time period, we need to find the corresponding SWEPAM data.

We use the ACE/SWEPAM data to conduct ballistic backmapping of the streamer/slow wind, a process by which we trace the in-situ measurements to their footpoint origins on a source surface defined at 2.5 solar radii (following the process described in works such as Zhao, Zurbuchen, and Fisk 2009 or Simunac et al. 2009). In ballistic backmapping, it is assumed that the solar wind propagates in a radial manner at a constant speed from the source surface to the in-situ spacecraft. We use 2.5 solar radii instead of one solar radius because this traditional source surface location in potential field source-surface models is where we can safely assume the magnetic field and solar wind to start to propagate only radially. Using SWEPAM data, we account for solar rotation in our backmapping by following the process laid out in Hefti et al. (2000):

6 $$ \lambda _{\mathrm{S}} = \lambda _{\mathrm{ACE}} + \Omega \Delta r/v, $$

where $\lambda _{\mathrm{S}}$ is the estimated heliographic longitude origin of the solar wind data of the source surface, $\lambda _{\mathrm{ACE}}$ is the heliographic sub-ACE longitude when the measurement was taken, $\Omega $ is the solar angular speed, $\Delta r = 0.98$ AU is the distance between the source surface and ACE, and $v$ is the proton bulk speed as measured by SWEPAM. The source-surface origin latitude is given as the latitude of ACE at measurement (i.e., the latitude of ACE’s footpoint at the source surface).

**Figure 5:**
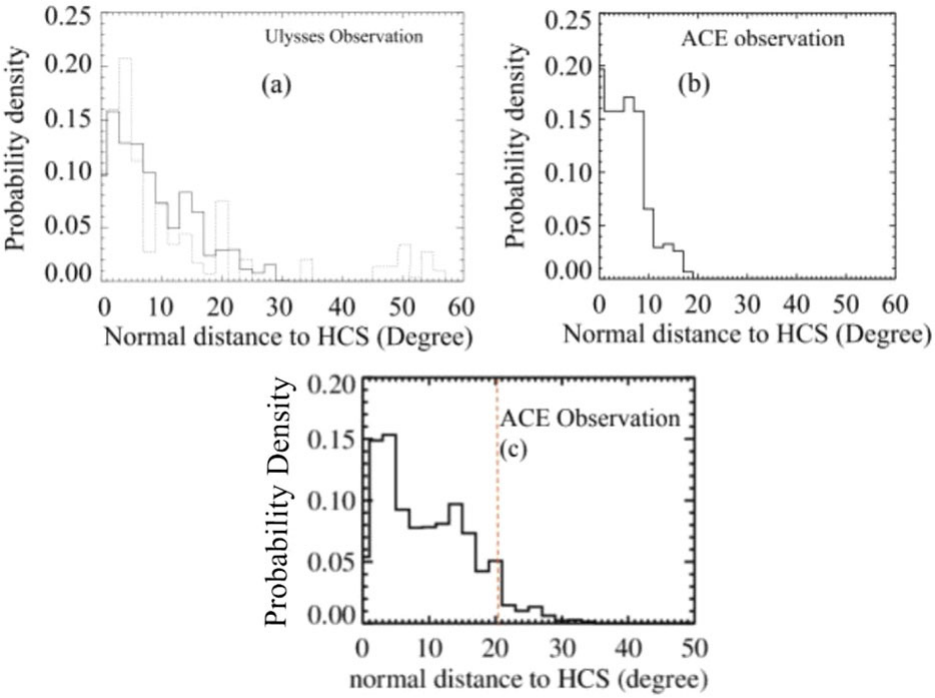
Probability density of streamer wind footpoints vs. normal distance to HCS used to measure streamer belt width in the minima of Cycles 23 (a) Zhao and Fisk (2011), 24 (b) Zhao and Fisk (2011), and 25 (c).

From here, we calculate the perpendicular or normal distance from the footpoints of the solar wind measurements we just found to the HCS, following the method laid out in Zhao and Fisk (2011). This is done using Stanford WSO data for the HCS locations for each Carrington rotation in a given solar minimum interval (wso.stanford.edu). Plotting the normal distance to the HCS allows us to constrain a half-width for the HCS streamer belt during a given minimum. We calculated the probability density of observing various angular normal distances from the HCS plotted this in Figure 5. Figures 4(a) and 4(b) show the results of this analysis from Zhao and Fisk (2011) for comparison, while Figure 5(c) shows the newest analysis for Cycle 25. In all three plots, we see that the probability density of observations drops off quickly as we extend outward from the HCS. At some point the probability that an observation belongs to this region becomes so low that we can safely define a cutoff point. We set this cutoff point across all minima to be at latitude where 95% of the streamer wind data falls within it. This is a relatively arbitrary cutoff point, but works well for all minima in identifying outliers and ensuring we are capturing the vast majority of the streamer wind in our measurements. For Cycle 25, we find the cutoff point in Figure 5(c) as being approximately 20 degrees normal from the HCS. Therefore, we can estimate that the width of the streamer belt itself during the Cycle 25 solar minimum is approximately 40 degrees. This value can now be used to directly calculate the solid angle of the non-streamer region.

### Measuring the Width of the HCS-Streamer Belt for Solar Cycle Minimum 22

One of the key observations needed to measure the width of the HCS-streamer belt at any given solar minimum is charge-state composition data. Without this data, we are unable to separate streamer from non-streamer wind and, thus, unable to narrow down a width for the HCS-streamer belt. Unfortunately, given that the earliest measurements of the charge state composition ratio of our O$^{7+}/$O^6+^ are from the 1990s, we would normally only be restricted to making measurements of the TOMF from Solar Cycle 23 and beyond.

Recently, however, Nakhleh et al. (2026) introduced a novel way to correlate in-situ measurements of proton-specific entropy to the charge state ratio of O$^{7+}/$O^6+^. They show that entropy in the solar wind, which is expected to be generated during plasma during heating in the corona, has variability that is specific to the source of the plasma and continues up to the freeze-in point of the plasma. When the plasma freezes-in, the entropy variability stops evolving and thus remains preserved from the source, much like charge state ratios. We leverage this result to measure the TOMF for the minimum of Solar Cycle 22 by utilizing entropy as a proxy for $\mathrm{O^{7+}/O^{6+}}$. This is particularly useful for us because entropy measurements only require measurements of plasma temperature and density. These are parameters that are readily available to study, even on missions without composition instruments. It should be noted that we cannot use this method prior to Solar Cycle 22 to find the TOMF not because there are no entropy measurements prior to Cycle 22, but because there are no available PFSS results modeling the HCS itself prior to Cycle 22.

Entropy has previously been used to identify solar wind stream interfaces Burton et al. (1999), and the correlation between solar wind entropy and the O$^{7+}/$O^6+^ charge-state ratio is robust across most solar wind conditions, particularly during solar minimum when the HCS and streamer belt are well defined. Although this correlation can break down during periods of low entropy (often associated with depleted carbon and active region-sourced wind streams near solar maximum), these regimes are not typical for the streamer belt and HCS wind at solar minimum. As such, entropy serves as a reliable proxy for charge-state composition in identifying the location streamer belt and HCS during solar minimum, with any correlation breakdowns having minimal impact on this analysis.

Following the derivations in Nakhleh et al. (2026), we define entropy as

7 $$ S = R\ln \left (\frac{R^{3/2}}{m_{\mathrm{p}}}\right ) + \frac{3R}{2}\ln \left (\frac{T_{\mathrm{p}}}{n_{\mathrm{p}}^{2/3}}\right ), $$

where $S$ is entropy, $T_{\mathrm{p}}$ is proton temperature, $n_{\mathrm{p}}$, is proton density, $m_{\mathrm{p}}$ is proton mass, and $R= k_{\mathrm{B}}/m_{\mathrm{p}}$ is the gas constant for protons (where $k_{\mathrm{B}}$ is Boltzmann’s constant).

**Figure 6:**
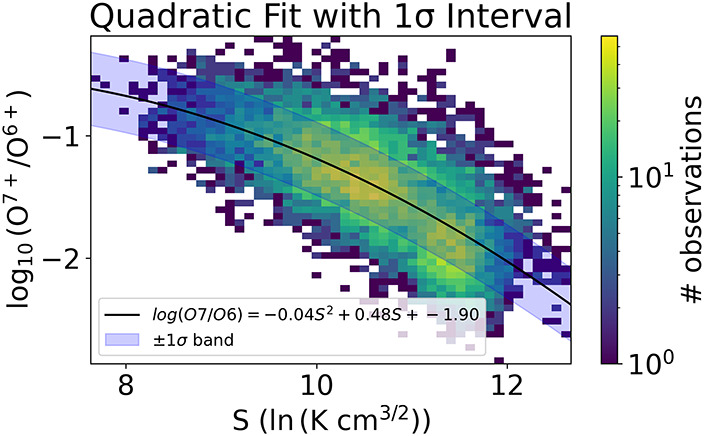
Quadratic fit with a $1 \sigma $ “prediction band” around the fit showing the fit relationship between the charge state ratio of O$^{7+}/$O^6+^ and entropy. This fit was created using over 11,000 data points taken at 1 AU.

We make use of ACE/SWICS and ACE/SWEPAM data from 2008 – 2010 here and make a quadratic fit to the data to create a relationship between O$^{7+}/$O^6+^ and entropy. This fit is created using data at solar minimum. Given that the solar minimum period from cycle to cycle is relatively unchanging, the fit we create with this data can be used at minimum from cycle to cycle as well. This fit can be seen in Figure 6.

**Figure 7:**
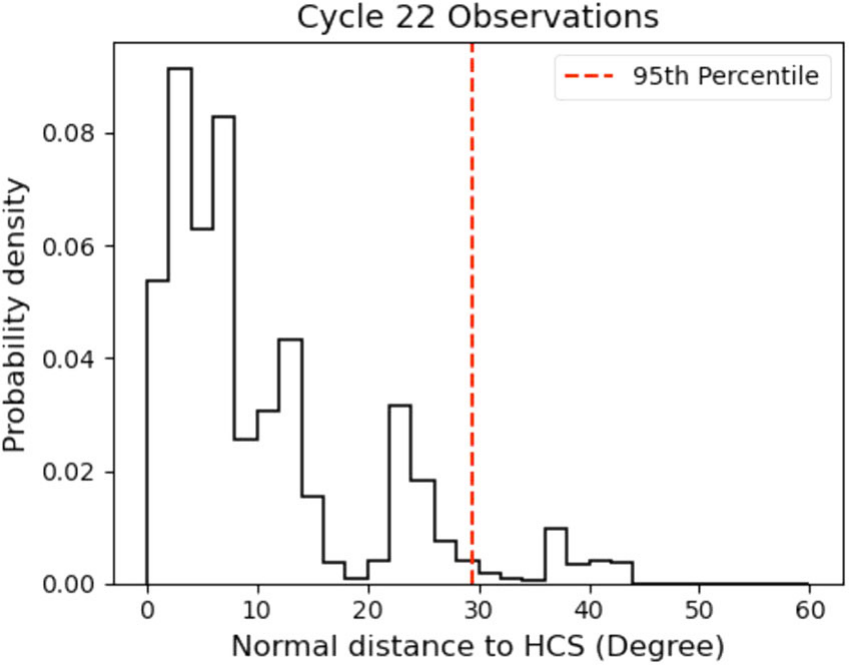
Probability density of streamer wind footpoints vs. normal distance to HCS used to measure streamer belt width in the minimum of Cycle 22.

The number of data points within the one sigma band for the quadratic fit is 69.6%. This is a good indicator that the fit is working because one would expect 68% of the data points to lie within 1 sigma for a normal statistical sample. Unfortunately the $R^{2}$ value for the linear fit is 0.48, and the quadratic fit $R^{2}$ is 0.49. This fit was created using over 11,000 data points, and the solar wind is inherently noisy, so it’s not completely unexpected, but these lines of best fit are certainly not perfect. However, for our purposes, this is still a usable relationship. Using this relationship to calculate O$^{7+}/$O^6+^, we can find TOMF in the same way as before. Figure 7 shows the probability density plot (like Figure 5) for the Cycle 22 solar minimum of the normal distance of streamer wind measurements to the HCS. We find that 95% of the streamer wind falls within a cutoff latitude of approximately 30^∘^.

## Conservation Model Results and Discussion

### Validation Results

In Section 1, we describe the new model for the conservation of TOMF that takes the form of Equation 5 as described by Zhao and Fisk (2011). Here, we find the TOMF for the solar minimum of Solar Cycle 25 according to this theory.

Using the methods described to find $\Omega _{\mathrm{OSB}}$ in Section 2.2, we find that the solid angle of the region outside the streamer belt during the Cycle 25 solar minimum was 1.14 steradians. We switch from degrees to steradians here because the above degree measurement was used for a distance and here steradians are a more interpretable area unit. Using the methods described to find $|B_{r}|r^{2}$ in Section 2.1, and as shown in Table 1 and Figure 3, across multiple spacecraft, we find that the open magnetic flux across the Cycle 24 – 25 solar minimum was approximately 2.81 nT AU^2^.

In the work of Zhao and Fisk (2011), the results of the new TOMF term at solar minimum for Cycles 23 and 24 are presented as relative ratios with the results of the Cycles 23 acting as a baseline (*i.e.,* $\frac{\text{Cycle 23 result}}{\text{Cycle 23 result}}$ and $\frac{\text{Cycle 24 result}}{\text{Cycle 23 result}}$). This means that we would expect to see that the relative ratios of the TOMF terms are always unity. We continue this trend with our results for the Cycles 22 and 25 solar minima, where we find the relative ratio of TOMF to be close to one. This discrepancy of being close to one, but within a reasonable margin of error, was also found for the Cycle 24 minimum in Zhao and Fisk (2011), so we can be confident that our findings are accurate and physical. Therefore, we safely claim that the idea that the new term TOMF is conserved from solar minimum to solar minimum has been validated for another solar minimum. The results of our observations and measurements in this study, as well as the same measurements found for previous cycles in Zhao and Fisk (2011) for the sake of comparison, are shown in Table 2.

**Table 2:** The results of confirming that the TOMF from solar minimum to solar minimum is a conserved quantity. The parameter values for the Cycles 23 and 24 minima come from Zhao and Fisk (2011), whereas the 22 and 25 results are the newest validations of the conservation theory for the Cycle 22 and 25 solar minima, respectively.

Cycle Minimum	Streamer Half-width (deg)	Non-streamer-belt Region Solid Angle ($\Omega _{OSB}$)	Relative Ratio of $|B_{r}|r^{2}$	Relative Ratio of Total Open Magnetic Flux ($\Omega |B_{r}|r^{2}$)
22	30	0.87	1.25	≈1
23	25	1	1	1
24	10	1.43	0.74	≈1
25	20	1.14	0.87	≈1

Table 2 shows the critical parameters in calculating the TOMF for the solar minima of Solar Cycles 22, 23, 24, and 25. Column 2 shows the distance measurement in degrees that is the streamer belt half-width at solar minimum. Column 3 takes that half-width measurement and uses it to calculate the solid angle of the non-streamer-belt region. Column 4 is the relative ratios of the open magnetic flux values from Table 1 against the open magnetic flux of Cycle 23 (*i.e.,* $\frac{(|B_{r}|r^{2})_{22}}{(|B_{r}|r^{2})_{23}}$, $\frac{(|B_{r}|r^{2})_{23}}{(|B_{r}|r^{2})_{23}}$, $\frac{(|B_{r}|r^{2})_{24}}{(|B_{r}|r^{2})_{23}}$, $\frac{(|B_{r}|r^{2})_{25}}{(|B_{r}|r^{2})_{23}}$). Column 5 is the same, except for the TOMF described by $\Omega |B_{r}|r^{2}$.

Converting the TOMF values at each minimum into units of a more usable flux value (*i.e.* nT AU^2^ to Mx), we find that the TOMF at solar minimum must always be approximately $5.1 \times 10^{22}$ Mx.

### Implications

**Figure 8:**
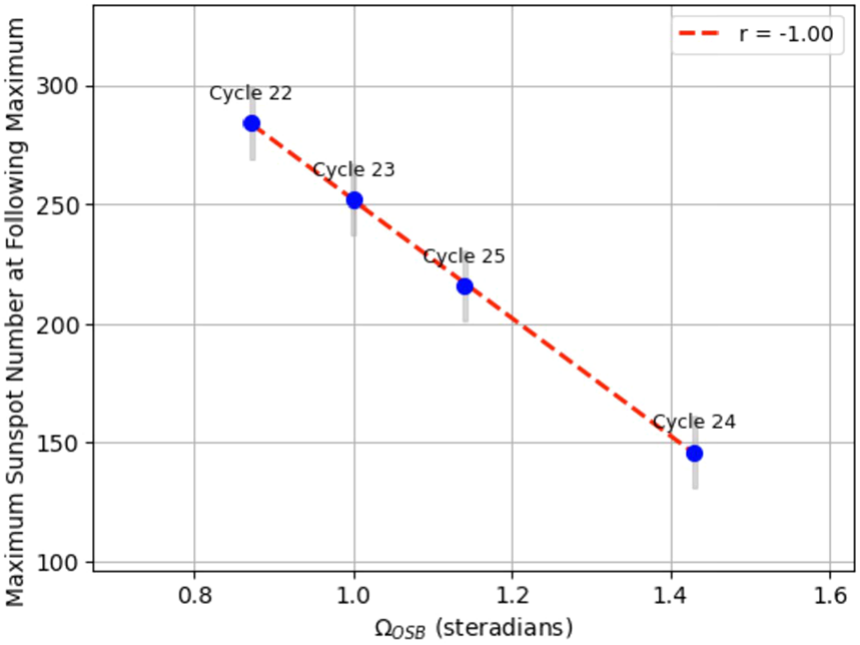
Relationship between the $\Omega _{\mathrm{OSB}}$ at cycle minima versus the maximum sunspot number at the following corresponding solar maxima, along with a standard linear regression. The sunspot data is monthly averaged here, so we estimate an error bar of 30 in the sunspot number to account for any lost information in the averaging, represented by the vertical gray bars. This new way of predicting the strength of a following solar cycle is similar to the field’s preferred method established in studies like Schatten et al. (1978) and Wang (2024) that use the strength of the radial polar magnetic field at solar minimum as direct indicators of the strength of the proceeding solar cycle. But whereas these studies make use of the field strength only, we take a step further and directly look at the region where the magnetic field is being generated at solar minimum, which we show here to be a very strong new predictive method.

The strong anti-correlation between Maximum Sunspot Number at the following maximum and the solid angle of the non-streamer belt region ($\Omega _{\mathrm{OSB}}$) seen in Figure 8 shows potential for predicting the next solar cycle. This direct inverse relationship makes sense and is supported by the correlation coefficient calculated through linear regression being −1.00. When the $\Omega _{\mathrm{OSB}}$ region has a bigger solid angle, the TOMF, still a constant amount at solar minimum, becomes more spread out. In turn, this makes it harder to form polar coronal holes and leads to a weaker solar cycle. When the open magnetic flux is spread out more, it can suppress or limit the formation and visible expansion of polar coronal holes. This is especially important during solar minimum, when large-scale changes or the spread of magnetic flux outside traditional coronal holes can cause this redistribution (Gilbert, Zurbuchen, and Fisk 2007). In turn, weaker and more disrupted polar field formation—due to non-ideal magnetic flux distribution—leads to reduced toroidal field generation and thus a weaker solar cycle (Jiang, Cameron, and Schuessler 2015).

As a concrete example, take the Cycle 24 minimum value of $\Omega _{\mathrm{OSB}} = 1.43$ steradians and note the height of the peak of the sunspot numbers (*i.e.*, the strength of the following solar cycle), 146 sunspots, in Figure 8. The following value of $\Omega _{\mathrm{OSB}}$ during the Cycle 25 minimum is a smaller value, 1.14 steradians, but it is clear that, even with the peak of the following solar cycle not yet reached, said solar cycle is stronger than the one before it - the time series data ends with 172 sunspots. This relationship works when including all four cycles worth of data into the mix. This indicates that $\Omega _{\mathrm{OSB}}$ can be used as a direct predictor for the strength of the solar cycle.

Figure 8 also helps us to validate the method we used to find the width of the HCS-streamer belt for the Solar Cycle 22 minimum. Even without the Cycle 22 data point, we would expect the linear relationship to hold true. The fact that the relationship holds with the addition of this point tells us that, despite the low $R^{2}$ value of the quadratic fit used to create the composition-entropy relationship, it still works well enough for us to accurately calculate TOMF.

## Concluding Remarks

This study builds upon the foundational work of Zhao and Fisk (2011), reinforcing the application of their conservation model to solar magnetic flux transport. By applying the conservation rule articulated through Equation 5, we affirm that the relative ratios of the TOMF consistently approach unity during the solar minima of Cycles 22, 23, 24, and now 25. Our analysis indicates that the TOMF at solar minimum remains approximately $5.1 \times 10^{22}$ Mx, thereby validating the robustness of this model in recent solar activity. Additionally, this result has led to strong evidence that the value of $\Omega _{\mathrm{OSB}}$ at solar minimum can predict the strength of the following solar cycle.

Despite the compelling nature of these results, our study is not without limitations. The choice of our intervals centered around the solar minimum is both arbitrary and contentious. Previous works, such as those by Harvey and White (1999) and Chapman and Dudok de Wit (2024), highlight the variability inherent in defining solar minima. It is imperative for future research to refine this temporal boundary to ensure more accurate measurements of TOMF.

In summary, our findings underscore the importance and validity of the conservation rule in modeling solar magnetic phenomena, while also setting a clear direction for future research endeavors. By addressing current methodological limitations and exploring more sophisticated modeling techniques, future studies can build upon the foundation laid by our research to advance our understanding of solar cycle processes.

## Data Availability

We used data from the Advanced Composition Explorer (ACE), Geotail, International Sun-Earth Explorer-3 (ISEE-3), Explorer 50 (IMP-8), Wind, and Solar Orbiter spacecraft publicly available on CDAWeb (https://cdaweb.gsfc.nasa.gov/).
